# Jefferson Medical College

**Published:** 1843-04-15

**Authors:** H. T. Child


### CLINICAL LECTURES AND REPORTS.

### JEFFERSON MEDICAL COLLEGE.

CLINIC OF PROFESSOR MÜTTER.

*Dispensary of Jefferson Medical College, Jan. 25, 1843.*

(Reported by H. T. Child.)

LECTURE IV.

Case IV.—*Ranula—Operation.*—Dr. M. remarked that the disease with which this patient, a female, aged 15 years, is affected, has been termed *Ranula*,* from the fancied resemblance of the tumour which characterises the complaint to the belly of a frog; or, according to some, from the fact that the voice is rendered by it hoarse or croaking. The anatomy, as well as the functions of the organs concerned in this disease, were but little understood by the older authorities, and hence it appears that ranula was often confounded with other affections. For instance, Celsus describes as ranula what must have been a common encysted tumour. Ætius supposed it to be nothing more than a varicose condition of the sublingual veins. Aranzi thought it a common abscess. Fabricius and Dionis considered it an encysted tumour of the melicerous kind. While Abul-Kasem, and the Arabians generally, viewed it as a malignant or cancerous affection. Louis, of Paris, was the first to indicate its true character, and the disease was shown by him to be nothing more than "a tumour containing fluids of different kinds, in which solid matter was occasionally found, and which originated from obliteration, either partial or complete, of the excretory ducts of the submaxillary and sublingual glands." It appears that the disease is confined to these sublingual ducts—those of the parotid and pancreas being too dense and unyielding to admit of the distension requisite to form a tumour resembling that found in ranula.

Although the definition of Louis is generally received as one correctly indicating the nature of ranula, we yet find some difference of opinion among modern surgeons relative to the precise mode of origin of this complaint. Dupuytren, for instance, states that up to this time it has not been clearly shown, by dissection or otherwise, whether the complaint is in reality situated in the sublingual ducts, or whether it consists merely in a serous cyst, or whether it may not originate in the dilatation of a mucous follicle. He, as well as Malgaigne and Breschet, believe that many cases reported as dilatation of the sublingual ducts are nothing more than serous cysts. Admitting the possibility of such mistakes, we shall yet consider ranula as a tumour, filled in its commencement with fluid, saliva or mucous, and originating exclusively in the obliteration of the salivary ducts of the sublingual and submaxillary glands.

From the experiments of Gmelin, however, it would appear, that in some cases after the disease has existed for a short time, the fluid is essentially different from saliva, containing no sulphocyanate, and only a small quantity of salicine, with some albumen. The albumen amounts to only two per cent., though the fluid is as thick as others containing about five per cent. It is supposed, therefore, "either that the fluid of ranula contains some peculiar material to which it owes its thickness, or else that the albumen in it is of some peculiar kind."

There is usually no difficulty in recognizing the disease—especially in its early stages. The tumour is either oval, or round, or lobulated; situated on one or both sides of the frænum linguæ; usually transparent, so that the nature of its contents may be detected at once; elastic when fully distended, but sometimes yielding a distinct fluctuation; scarcely if at all painful; and rarely producing any inconvenience, unless it attains a large size, when it displaces the anterior portion of the tongue, turning it back upon the fauces, and thus occasioning difficulty in deglutition, as well as imperfect and indistinct articulation. When the tumour has existed for some time, its walls become thicker and less transparent, and its contents change in consistence, so that the diagnosis is more difficult. The fluid, at first, is nothing more than saliva or mucous, but it soon becomes of the consistence and color of white of egg; sometimes it is purulent; and often we find concretions of a calcareous or even stony nature; and in a case reported by Tulpius, the whole mass was composed of a dense hard tissue, and was removed by the application of the actual cautery. Whenever these changes, or any one or more of them take place, the disease may be confounded with tumours of different kinds occupying the location of ranula. In a case to which I was recently called by my friend Dr. McCleane, a steatomatous tumour, of the size of a small orange, occupied the precise spot of ranula; and from the sac's containing fluid sufficient to give rise to fluctuation, I was under the impression that it was nothing more than an example of salivary tumour, with its walls thicker than usual, and containing along with the fluid a portion of solid matter. In removing it, it was found necessary to dissect out a large sac nearly filled with a caseous substance, and the case was, in reality, one altogether different from ranula.

Although ranula cannot be considered an affection of much danger, we have cases reported by Hildanus, Marchettis, Alix, Taillardan, Allen Burns, Cline, Velpeau, Bonnet and others, in which, from the immense size of the tumour, the patients were in imminent danger of losing their lives from suffocation and compression of the carotid arteries. Cooper also cites cases where the tongue was much displaced, and mastication and deglutition materially interfered with. In infants it prevents the action of sucking.

*Causes.*—The closure of the ducts of the sublingual or submaxillary glands may be occasioned by inflammation of the tongue or its mucous coat, by apthæ, or common ulceration of the ducts themselves, by wounds of the ducts, or the operation for tongue-tie or stammering, and by the lodgement of calculous matter, or inspissated mucous or saliva in their orifices. Occasionally the disease is congenital, as the cases of Stoltz and Dubois clearly prove. Dupuytren and Breschet doubt this, and suppose that all such examples are in reality only serous cysts.

*Treatment.*—Ranula, although generally requiring an operation for its relief, is nevertheless susceptible, though rarely, it is true, of spontaneous cure. Sometimes a fistulous orifice may be formed in the walls of the tumour by ulceration; and occasionally, when the tumour acquires considerable size, it bursts, and its contents escaping, the sac is obliterated; in either case a cure may take place without the assistance of the surgeon. But inasmuch as the treatment is usually devoid of danger, and certain in its results, we should never trust to nature where a case is brought to us. We must relieve the patient as soon as possible.

The indications in the treatment are very simple. We must, in the first place, carefully remove the obstruction of the duct, provided this is practicable. If this cannot be done, we must establish a fistulous opening through which the saliva may escape, or we must obliterate the sac, by causing its inner sur-

* From Rana, a Frog.

face to granulate after its contents have been removed, or we may remove it.

The first indication is readily accomplished—provided the obstruction be calculous or inspissated mucous—by picking out the foreign body with the forceps, or by cutting it out where we cannot remove it by the forceps alone. When the obstruction is the result of chronic inflammation with thickening, we may sometimes overcome the difficulty just as we do in affections of a similar nature, involving other mucous cavities or tubes, by the repeated introduction of small bougies or probes, or by allowing small leaden stilets to remain for a day or two at a time in the duct, as advised by Louis. When the ducts are obliterated, we must resort to other means. The plan first tried was nothing more than making a simple incision into the sac, by which its contents were evacuated, and then leaving the case to itself. These incisions universally fail to afford relief, inasmuch as the cut soon heals, and then the integrity of the sac being restored, the fluid soon accumulates. In order to prevent the closure of the wound, it is proposed to make an oval or round incision, and then touch the margins with nitrate of silver, so as to render them callous. This plan, originating with Louis or Camper, is the method I prefer to all others, and will succeed in nearly every case of pure ranula. To accomplish the same end, it was long since proposed to introduce some foreign body into the wound—pieces of lint, a temporary style, hollow tubes, &c. &c.; but Dupuytren was the first to propose the introduction of something intended to remain until the cure was accomplished.

He used a little button, termed by him "bouton à demeure," (from the circumstance of its being left a long time in the wound,) consisting of two elliptical plates or buttons, five or six lines broad in their greatest diameters, and joined together by a pedicle or stem two or three lines in length; the external surface of these plates was convex, the internal concave. A puncture two lines long being made in the tumor, and its contents discharged, one button is introduced into the cyst, and the other remains in the mouth.

This plan, a modification of that of Lecat, will answer in some cases, but is not to be preferred to that of Louis or Camper. Various attempts have been made to destroy the sac, or cause it to granulate, some of which are occasionally useful; but they should never be performed where we can possibly get along with the more simple methods just described. The most ancient of these operations is probably that in which the sac is destroyed by *caustic* or *escharotics*, or the *actual cautery;* it is rarely, if ever, resorted to at the present day. Injecting the sac with some stimulating liquid, as we do in hydrocele, has also been employed, but with a success so partial, as scarcely to warrant our receiving the method among those deserving confidence. The introduction of a seton with the same view, has been recommended by Physick, Home, Laugier and others, and is unquestionably sometimes a successful means, but it causes great inconvenience, and is not so certain as the operation of Louis. Finally, when all other measures fail, and the tumor is large, it may be necessary either to excise a considerable portion of its walls, or dissect out the whole mass. The operation is dangerous, and should not be resorted to except in cases of absolute necessity.

Prof. M. then proceeded to operate by cutting out a fold of the wall of the tumour and evacuating the contents, which resembled white of egg. The parts were then touched with argentum nitratum. The operation was simple, and gave the patient little or no pain.

